# Circadian Syndrome and Stress Urinary Incontinence in Adult American Women: A NHANES Cross-Sectional Study

**DOI:** 10.5152/tud.2026.26028

**Published:** 2026-09-03

**Authors:** Yuan Zheng, Tuochen Lv, Shichao Dai, Tita Tadoh Mike Pleass, Muhammad Arslan Ghaffar, Sisi Lu, Hai-Hong Jiang

**Affiliations:** The First Affiliated Hospital of Wenzhou Medical University, Wenzhou, China

**Keywords:** Circadian syndrome, National Health and Nutrition Examination Survey, stress urinary incontinence

## Abstract

**Objective:**

The circadian syndrome (CircS) is linked with chronic diseases such as gallstones, kidney stones, and overactive bladder. However, the relationship between CircS and stress urinary incontinence (SUI) is poorly understood. The study aimed to investigate the association of CircS and SUI by analyzing data from the National Health and Nutrition Examination Survey (NHANES) 2007-2018.

**Methods:**

The cross-sectional analysis was based on the 2007-2018 NHANES. Data on SUI were collected from the questionnaire. Three multivariate logistic regression models were used to elucidate the association between CircS and SUI prevalence. Stratified and interaction analyses were conducted to identify factors that could modify this relationship.

**Results:**

A total of 5758 female patients were included in the study. In the fully adjusted model, patients with CircS showed a significantly higher prevalence of SUI (odds ratio [OR] = 1.250, 95% confidence interval [CI], 1.033-1.513) compared to the non-CircS group. Additionally, there was no significant correlation between the number of CircS components and the risk of SUI. The association between these factors was modified by an interaction with age (*P* < .001).

**Conclusion:**

There is a positive correlation between CircS and SUI prevalence. These findings provide new perspectives for future intervention strategies. However, further research is needed to explore its underlying biological mechanisms.

**Main Points**

- There is a positive correlation between circadian syndrome (CircS) and stress urinary incontinence (SUI).
- The study is representative and applicable based on National Health and Nutrition Examination Survey (NHANES), a national multi-ethnic survey of a large population.
- CircS is a new concept that combines circadian rhythms and is more consistent with most physiological processes.

## Introduction

Urinary incontinence (UI) is a pathological condition that adversely affects many people, and stress urinary incontinence (SUI) is a common subtype characterized by involuntary leakage during activities such as sneezing and coughing.^1^^,^^2^ Stress urinary incontinence is more common in women and is associated with bladder or pelvic floor muscle dysfunction.^3^ The prevalence of SUI peaks in middle-aged women and then declines.^4^ The pathological mechanism of SUI is associated with age-related muscle mass and neurodegeneration, as well as muscle dysfunction.^5^

Circadian rhythms are produced by the internal body clock, which consists of a central clock in the brain and peripheral clocks in the organs. These rhythms are produced through transcription factors and gene expression and regulate key physiological functions in the body, such as metabolism.^6^^-^^8^ The body clock also regulates peripheral body clocks that exist in nearly every organ in the body, such as the liver, heart, muscle, and fat tissue.^9^ It must be emphasized that the circadian rhythm system plays a pivotal role in human health and metabolism.^10^ Some unhealthy lifestyles, including sleep disorders, use of artificial light, and shift work, have been documented to cause circadian rhythm disruption, which has adverse effects on human health.^11^ In recent years, research has increasingly focused on the correlation between circadian rhythm systems and chronic diseases.^12^^,^^13^ Circadian syndrome includes any 4 of the following conditions: dyslipidemia, elevated triglycerides or reduced high-density lipoprotein cholesterol (HDL-C), hypertension, central obesity, diabetes, depression, and sleep deprivation. When people remain in an unhealthy state for an extended period, their circadian rhythms become vulnerable and more susceptible to disruption.^11^

Recent studies have demonstrated that metabolic syndrome (MetS) and its comorbidities (sleep deprivation and depression) significantly influence the prevalence of SUI. Furthermore, growing evidence indicates that CircS is associated with cardiovascular disease (CVD), lower urinary tract symptoms, and kidney stones.^14^^-^^16^ All these factors are not only closely related to the internal circadian clock, but also risk factors for SUI. Therefore, it is worth exploring whether there is a relationship between CircS prevalence and SUI. To the authors’ knowledge, no studies have been conducted on the association between CircS prevalence and SUI. Therefore, this cross-sectional study was established by analyzing extensive population data from the NHANES. The authors hypothesized that CircS prevalence would show a positive correlation with SUI prevalence.

## Material and Methods

### Research Design and Population

All data analyzed here were cited from the January 1, 2007-December 30, 2018 NHANES database, conducted by the US Centers for Disease Control and Prevention (CDC). This database primarily consists of a series of cross-sectional, nationally representative surveys involving civilian, non-institutionalized American adults, designed to measure the health and nutritional status of American adults and children.

Ethical committee approval was received from the Research Ethics Review Board of NCHS (Approval No.:Continuation of Protocol #2005-06, Continuation of Protocol #2005-06, Protocol #2011-17, Continuation of Protocol #2011-17, Continuation of Protocol #2011-17, Protocol #2018-01 Effective beginning October 26, 2017; Date: 2007-2008, 2009-2010, 2011-2012, 2013-2014, 2015-2016, 2017-2018). Written informed consent was obtained from the participants who agreed to take part in the study.

### Diagnosis of Stress Incontinence

This study is a retrospective analysis. The study assessed SUI using self-reported questionnaires. Researchers asked participants: “Did you experience any leakage during physical activities such as coughing, lifting weights, or exercising?” A positive response indicated the presence of SUI.

### Diagnosis of Circadian Syndrome

As the primary exposure variable, CircS was diagnosed when a person has the following ≥4 components: (1) central obesity: women (men) with waist circumference ≥88 cm (≥102 cm); (2) elevated triglycerides (≥ 150 mg/dL) or use of lipid-lowering drugs; (3) HDL-C reduction (men <40mg/dL, women <50mg/dL) or use of lipid-lowering drugs; (4) elevated blood pressure (systolic ≥ 130 or diastolic ≥ 85 mmHg) or use of antihypertensive drugs; (5) fasting blood glucose elevation (≥100 mg/dL) or use of antidiabetic drugs; (6) reduced sleep time (≤6 hours/day); and (7) depressive symptoms defined by patient health questionnaire-9.

### Covariates

Covariates were collected through questionnaires, clinical evaluations, and laboratory tests. Continuous variables included age (≥ 20 years), poverty income ratio (PIR), body mass index (BMI), serum uric acid (SUA), and creatinine (Scr). The categorical variables were classified as race (non-Hispanic black, non-Hispanic white, other Hispanic, Mexican American, and others), educational level (less than high school, high school or above), marital status (married, unmarried), smoking, alcohol, cancer, stroke, coronary heart disease (CHD), and vigorous activity (yes or no).

### Statistical Analysis

Proportions and averages (standard errors) were used to represent baseline characteristics. Categorical variable comparisons were adjusted using chi-square analysis, and *t*-tests were used to compare continuous variables.

Participants were divided into 2 groups according to SUI diagnosis, and 3 multivariate logistic models were established to analyze the association between CircS and SUI. In addition, the correlation between the number of CircS components and SUI was analyzed. In the unadjusted model, no adjustment factors were included. In the minimally adjusted model, age and race were adjusted. For the fully adjusted model, age, race, education, BMI, marital status, PIR, smoking, vigorous activity, alcohol, stroke, CHD, SUA, Scr, and cancer were adjusted. In order to further analyze the association, stratification and interaction analysis were also performed for all of the above potential confounding factors.

Considering the complex probability clustering design of NHANES, this study incorporated weights into the statistical analysis. All statistical analyses were performed using R software, version 4.4.2 (R Foundation; Vienna, Austria). A *P*-value < .05 is considered statistically significant.

This study did not use AI and/or LLM tools during the preparation of this manuscript.

## Result

### Baseline Demographic Characteristics

**Figure 1:**
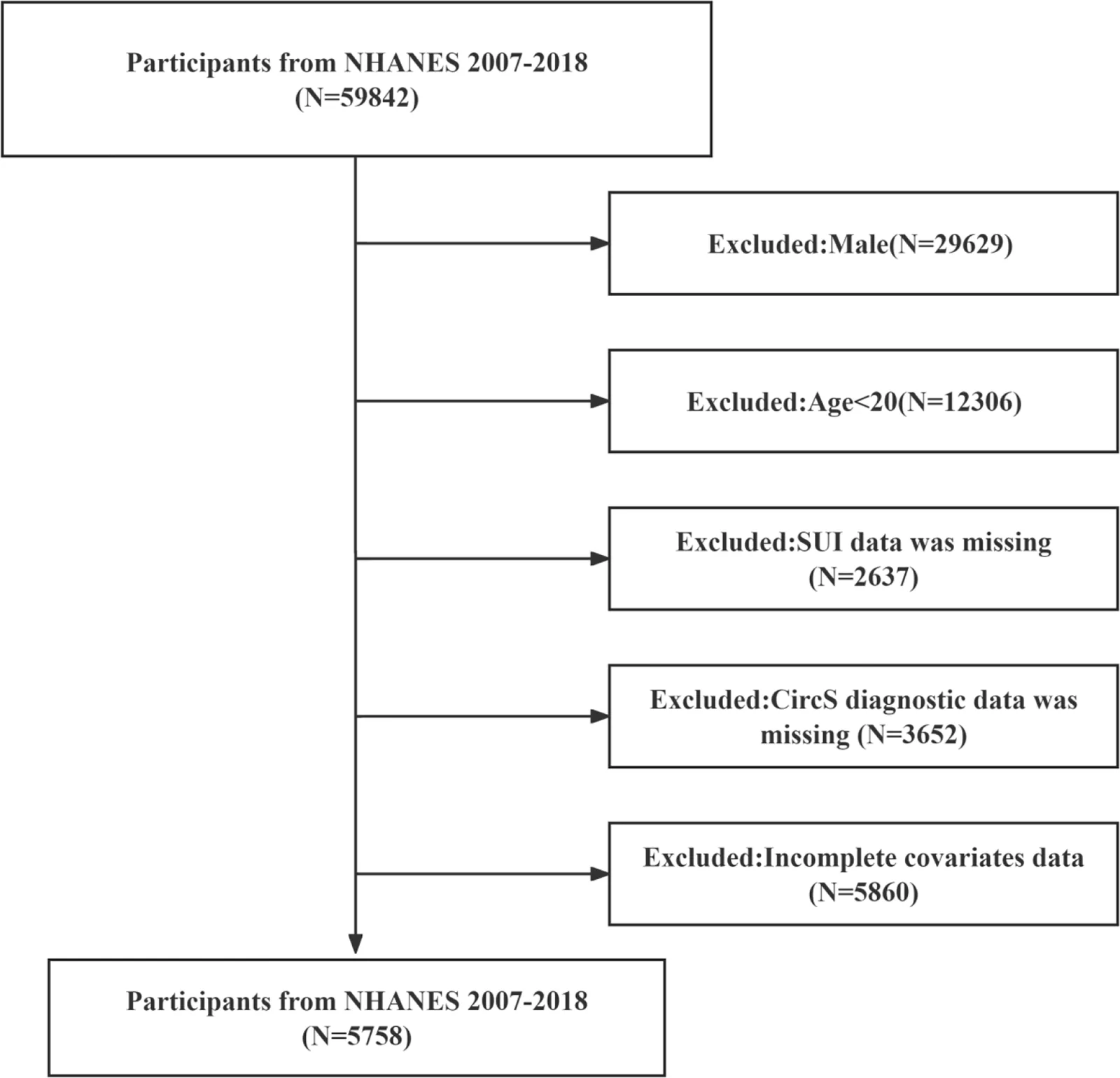
Flow diagram of obtaining the final inclusion in the population.

From the NHANES 2007-2018 cycle, a total of 5758 participants were eligible, and the specific inclusion and exclusion criteria are shown in Figure 1. Most baseline characteristics were statistically significantly different between groups classified by SUI. As shown in Table 1, SUI patients were older (average (SD) = 52.8 (15.8) years old) and tended to have a higher BMI (30.83 (7.53) kg/m^2^) and SUA (298.3 (77.7) μmol/L). In addition, the prevalence of CircS in this group was significantly higher (44.2%). In addition, compared with the non-SUI group, patients with SUI were more likely to be married (58.7%), smoking (41.1%), CHD (3.3%), cancer (12.0%), and stroke (4.9%). However, in the SUI group, patients who engaged in vigorous activities (14.4%) and had a high level of education(76.0%) were less common than non-SUI group. No significant differences in PIR and alcohol status were found between the 2 groups.

### Association Between Circadian Syndrome and Prevalence of Stress Urinary Incontinence

Table 2 shows the association between CircS and SUI prevalence. The results showed that the risk of SUI was higher in CircS patients than in non-CircS patients (model 1: OR = 2.058, 95% CI: 1.773-2.387). Similarly, after additional adjustment for confounders (model 3), the association between CircS and SUI was stable, indicating an increase in the prevalence of SUI by 25% (OR = 1.250, 95% CI: 1.033-1.513).

In addition, there was no significant correlation between the number of CircS components and the risk of SUI.

### Layering and Interaction Analysis

**Figure 2:**
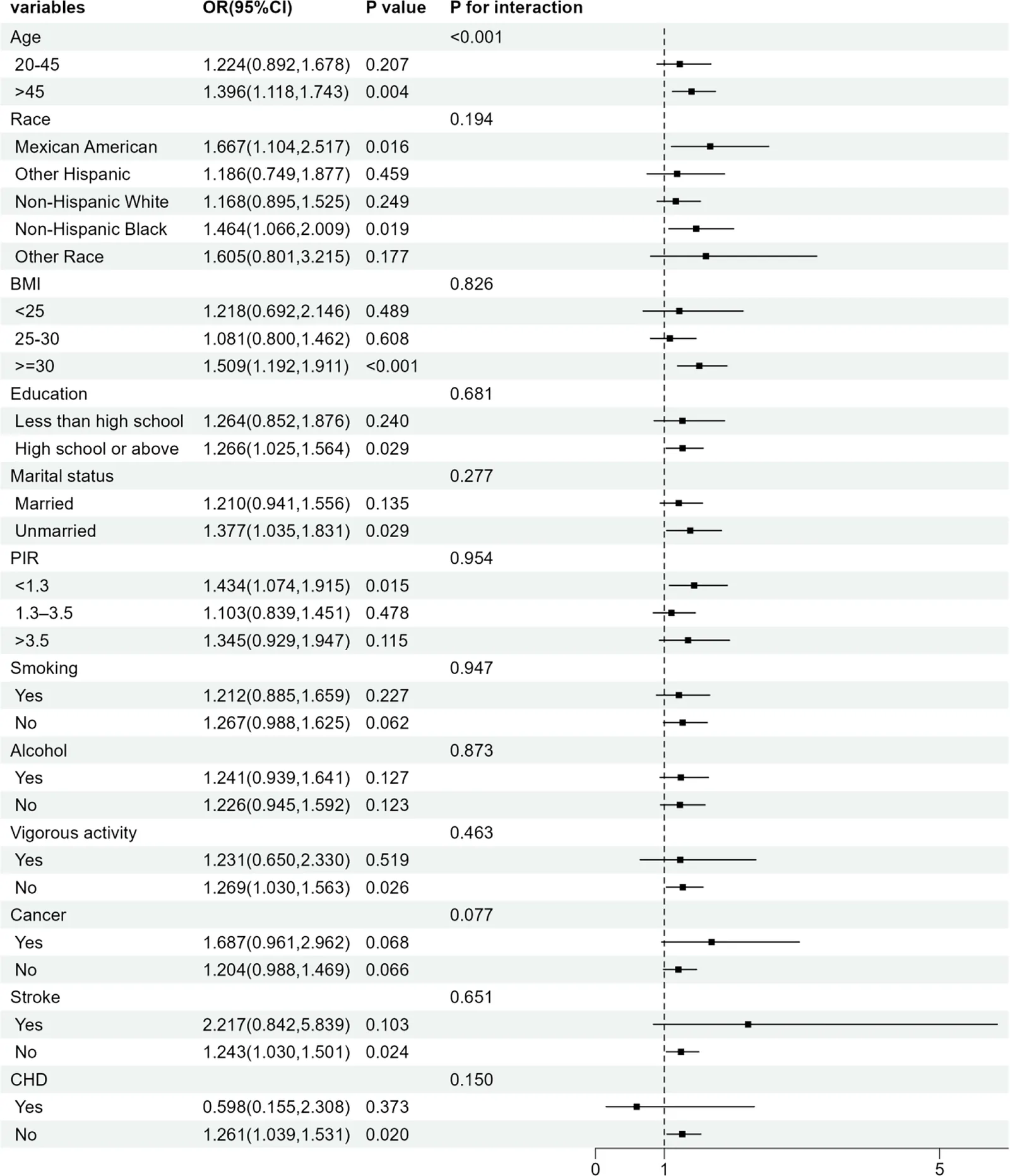
Subgroup analysis for the association between circadian syndrome (CircS) and stress urinary incontinence (SUI). Age, race, education, body mass index (BMI), marital status, poverty income ratio (PIR), smoking, alcohol, vigorous activity, coronary heart disease (CHD), stroke, and cancer were adjusted, except for the corresponding variables.

To further clarify the association between CircS and SUI prevalence, the authors performed an interaction analysis in a fully adjusted model (Figure 2). The authors found that the association between the 2 was altered by an interaction with age (*P* < .001). Furthermore, the positive correlation was more pronounced when people were in the 20-45 or >45 age group (OR: 1.224,1.396). However, the direction of effect values was consistent across subgroups, and no other factors showed interaction. In addition, it is worth noting that a significant positive correlation was observed between CircS and SUI prevalence in obese individuals (OR = 1.509, 95% CI: 1.192-1.911, *P* < .001).

## Discussion

This study showed a positive correlation between CircS and SUI prevalence, and no significant correlation was found between the number of CircS components and the risk of SUI. In addition, the positive association changed with age, but the direction of the effect values is consistent across subgroups.

Although substantial research has been conducted on the association between SUI and MetS, depression, and sleep deprivation, these factors have always been analyzed separately rather than considered together. This is why the first study demonstrating the correlation between CircS and SUI is presented here. With the change of life rhythm and lifestyle, the prevalence of CircS is increasing year by year.^11^ Circadian syndrome is primarily dependent on circadian rhythm dysfunction, which is a set of biological variables (approximately 24-hour cycle) produced by the body clock.^17^ The circadian rhythm can not only affect the macro organism, but also affect a variety of organs and cells, making it one of the most important and basic characteristics of biological activities.^18^ When circadian rhythm is altered abnormally, there should be a number of problems in the body, including inflammation, immune and metabolic disorders. All of these changes contribute to the development of SUI.

Misalignment with environmental cues can disrupt circadian homeostasis, adversely affecting human behavior (sleep-wake cycles and rest-activity rhythms) and overall health. For instance, shift workers and travelers crossing multiple time zones are particularly susceptible to circadian desynchronization, which can initiate a vicious cycle contributing to a range of metabolic, endocrine, and cardiovascular disorders. These include obesity, insulin resistance, altered cortisol rhythms, and elevated blood pressure.^19^ Similarly, sleep deprivation, poor sleep quality, and narcolepsy have been shown to dampen the rhythmic expression of clock genes and are associated with an increased risk of diabetes, metabolic syndrome, and obesity. All of these factors are risk factors for SUI. As a potential countermeasure, appropriately timed exercise has been found to improve sleep quality and facilitate adaptation to shift work, thereby reducing negative metabolic outcomes. Furthermore, inverted sleep-wake cycles and insomnia represent significant risk factors for nocturia, underscoring the critical role of circadian dysregulation not only in disease pathogenesis, but also as a promising therapeutic target.

Stress urinary incontinence is directly related to diabetes and metabolic syndrome, and diabetes is the most important independent determinant of SUI.^20^ For women with relatively controlled diabetes, deterioration in glycemic control is more strongly associated with a higher likelihood of developing SUI.^21^ Metabolic syndrome increases the risk of SUI and may have an even greater impact on postmenopausal women.^22^ On the one hand, the peripheral neuropathy caused by metabolic syndrome can cause dysfunction of the nerves governing the organs or muscle weakness and atrophy, leading to urinary incontinence.^23^ Because pelvic floor neuromuscular injury is one of the important anatomical mechanisms of SUI,^24^ on the other hand, metabolic syndrome can lead to obesity, which is closely associated with the incidence of SUI.^25^ Obesity and related metabolic disorders are also associated with systemic inflammation, release of pro-inflammatory cytokines and oxidative stress, which alter collagen metabolism, accompanied by increased intra-abdominal pressure, leading to the progression of SUI.

With the deepening of research on urinary system diseases, the understanding of the relationship between sleep disorders (1 component of CircS) and SUI risk is deepening.^26^^,^^27^ Notably, 1 study indicates that among patients with overactive bladder (OAB), sleep disorders and fatigue were associated with more severe SUI and OAB symptoms, worse health-related quality of life, and worse social and mental health.^28^ The way sleep disorders lead to neurodegeneration and endocrine dysfunction is similar to aging, suggesting that sleep disorders may affect the incidence and severity of age-related chronic diseases (such as SUI).^29^

It is conceivable that the adverse effects of insomnia on mental health may affect SUI symptoms. Studies have shown that depression (also a component of CircS) is associated with the severity of SUI symptoms and dysfunction. In addition, the negative emotional effects of SUI can in turn lead to sleep disorders, because the association between insomnia and psychological distress is bidirectional.^30^

Serum uric acid differed significantly between SUI and non-SUI groups in Table 1. Obesity and metabolic syndrome are the key links. Obesity continuously increases abdominal pressure through fat accumulation, directly damaging the pelvic floor support structure, making it the primary risk factor for stress urinary incontinence; it is also one of the most common causes of hyperuricemia. The metabolic syndrome itself and its core symptom “increased fasting blood sugar” both significantly increase the risk of stress urinary incontinence in women.

The study also showed that age altered the positive correlation between CircS and SUI prevalence, but the direction of effect values was consistent across subgroups. According to the subgroup analysis, the positive correlation between SUI and CircS was more significant in patients older than 45 years old and obese people. Previous studies have reported that older adults are more susceptible to SUI, which is consistent with the results. Circadian rhythm disruption accelerates aging, and the aging process is associated with circadian rhythm vulnerability. These older adults, especially those who are overweight, are more likely to develop sleep disorders and psychological problems such as depression and anxiety. Therefore, the authors pay close attention to the physical and mental health of the older adults.

This study has both advantages and disadvantages. First, the study is representative and applicable based on NHANES, a national multi-ethnic survey of a large population. Second, CircS is a new concept that combines circadian rhythms and is more consistent with most physiological processes. No studies have focused on the association between CircS and SUI prevalence.

However, the study has some limitations. First, the SUI diagnosis is based on self-reporting, which may underestimate the prevalence of the disease. Second, although the authors controlled for many covariates to consider relevant confounding factors, there may still be residual and unmeasured confounding factors (such as dietary factors). Finally, the observations are focused on American populations, limiting the findings to other ethnic groups. Future research should consider larger and more diverse populations to validate the findings. In addition, in-depth research into the biological mechanisms between CircS and SUI will help develop more effective treatments.

Furthermore, because many individuals lacking data were excluded from the observational analysis (20%), there may be potential selection bias. Considering the observational nature of this study, such bias is inevitable. This bias may mainly stem from the loss of samples during the enrollment stage. To control for bias, the authors used multivariate regression models to adjust for known confounding factors. Although the authors tried the best to control for selection bias through design and statistical methods, due to the inherent limitations of observational studies, residual confounding or unmeasured selection bias may still exist. However, it does not affect the main conclusions of this study.

In summary, based on a large observational study of NHANES, the authors found that CircS was a strong predictor of increased risk of SUI. This finding holds significant importance in understanding the relationship between CircS and SUI, and provides a new perspective for future clinical intervention. Because cross-sectional design is limited, the findings need to be verified, and the underlying mechanisms should be further clarified.

There is a positive correlation between CircS and SUI. In addition, there is no significant correlation between the number of CircS components and the risk of SUI. However, in order to gain a deeper understanding, more comprehensive and prospective cohort studies are necessary.

## Figures and Tables

**Table 1.** Demographic and Clinical Characteristics of the Participants with and Without Stress Urinary Incontinence

Variables	Non-SUI	SUI	*P*-value
Number	3336	2422	
Age	46.6 (18.2)	52.8 (15.8)	<.001
Race (%)			<.001
Mexican American	442 (13.2)	408 (16.8)	
Other Hispanic	351 (10.5)	263 (10.9)	
Non-Hispanic White	1349 (40.4)	1182 (48.8)	
Non-Hispanic Black	805 (24.1)	356 (14.7)	
Other race	389 (11.7)	213 ( 8.8)	
Education level (%)			<.001
Less than high school	656 (19.7)	581 (24.0)	
High school or above	2680 (80.3)	1841(76.0)	
Marital status (%)			<.001
Married	1720 (51.6)	1421 (58.7)	
Unmarried	1616 (48.4)	1001 (41.3)	
BMI (kg/m2)	28.59 (7.37)	30.83 (7.53)	<.001
Circadian syndrome (%)			<.001
Yes	959 (28.7)	1071 (44.2)	
No	2377 (71.3)	1351 (55.8)	
Scr (μmol/L)	67.9 (28.6)	67.7 (22.4)	.713
SUA (μmol/L)	289.7 (76.9)	298.3 (77.7)	<.001
PIR	2.47 (1.63)	2.43 (1.61)	.399
Smoking (%)			<.001
Yes	1122 (33.6)	995 (41.1)	
No	2214 (66.4)	1427 (58.9)	
Alcohol (%)			.993
Yes	1850 (55.5)	1342 (55.4)	
No	1486 (44.5)	1080 (44.6)	
Vigorous activity (%)			<.001
Yes	695 (20.8)	349 (14.4)	
No	2641 (79.2)	2073 (85.6)	
CHD (%)			.008
Yes	70 ( 2.1)	79 ( 3.3)	
No	3266 (97.9)	2343 (96.7)	
Stroke (%)			<.001
Yes	99 ( 3.0)	118 ( 4.9)	
No	3237 (97.0)	2304 (95.1)	
Cancer (%)			<.001
Yes	256 ( 7.7)	290 (12.0)	
No	3080 (92.3)	2132 (88.0)	

For categorical variables, *P* values were analyzed by chi-square tests. For continuous variables, the *t*-test for slope was used in generalized linear models.

Chi-square detected the difference between non-SUI group and SUI group.

BMI, body mass index; CHD, coronary heart disease; PIR, poverty income ratio; Scr, creatinine; SUA, serum uric acid; SUI, stress urinary incontinence.

**Table 2.** Association Between Circadian Syndrome and Stress Urinary Incontinence

Variables	Model 1		Model 2		Model 3	
	OR (95% CI)	*P*	OR (95% CI)	*P*	OR (95% CI)	*P*
Circadian syndrome						
No	Reference		Reference		Reference	
Yes	2.058 (1.773,2.387)	<.001	1.615 (1.371,1.903)	<.001	1.250 (1.033,1.513)	.023
Components of circadian syndrome						
4	Reference		Reference		Reference	
5	1.125 (0.879,1.440)	.346	1.128 (0.879,1.449)	.340	1.081 (0.834,1.402)	.550
≥6	1.476 (1.036,2.103)	.031	1.515 (1.054,2.176)	.025	1.376 (0.928,2.039)	.111

Model 1 adjusted for: none. Model 2 adjusted for: age and race. Model 3 adjusted for: age, BMI, race, education level, marital status, Scr, SUA, PIR, smoking, alcohol, vigorous activity, CHD, stroke, and cancer.

BMI, body mass index; CHD, coronary heart disease; CI, confidence interval; OR, odds ratio; PIR, poverty income ratio; Scr, creatinine; SUA, serum uric acid; SUI, stress urinary incontinence.

## Data Availability

The data that support the findings of this study are available on request from the corresponding author.
